# Role of complete blood count in the diagnosis of culture-proven neonatal sepsis: a systematic review and meta-analysis

**DOI:** 10.1136/archdischild-2025-328523

**Published:** 2025-05-24

**Authors:** Emily Hyde, Mark Anthony, Stephen Kennedy, Manu Vatish

**Affiliations:** 1 Nuffield Department of Women’s and Reproductive Health, University of Oxford, Oxford, UK; 2 Newborn Care Unit, Oxford University Hospitals, Oxford, UK

**Keywords:** Haematology, Neonatology, Intensive Care Units, Neonatal, Sepsis

## Abstract

**Objective:**

Neonatal sepsis is a significant cause of morbidity and mortality, particularly in preterm infants. Despite its routine use in adults, the diagnostic utility of complete blood count (CBC) in neonatal sepsis remains debated. This systematic review and meta-analysis aimed to evaluate the diagnostic accuracy of CBC parameters for neonatal sepsis.

**Methods:**

This review was registered at PROSPERO (CRD42023476510). MEDLINE, Embase, CINAHL and the Cochrane Library were searched from database inception to 28 October 2024. Observational studies of neonates with sepsis, published in English, were included. Pooled diagnostic accuracy metrics were calculated for CBC parameters, including the white cell count (WCC), neutrophil count and immature-to-total neutrophil ratio (ITR). Bias was assessed using a modified QUADAS-2 tool.

**Results:**

Functional CBC parameters like ITR and mean neutrophil volume (MNV) showed moderate diagnostic accuracy. Pooled analysis revealed that an ITR >0.20 had 66.3% sensitivity and 85.4% specificity for neonatal sepsis. MNV also showed promising diagnostic utility, but substantial heterogeneity across studies (I^2^>0.80) limited its generalisability. Traditional parameters like the WCC and platelet count had lower diagnostic accuracy.

**Conclusions:**

The CBC is a rapid, cost-effective test requiring minimal blood volume, making it a practical adjunct in neonatal diagnostics. Functional parameters like ITR and MNV show the potential to complement existing approaches but are insufficient as stand-alone diagnostic tools. Further research is needed to validate their clinical utility and address heterogeneity in study designs.

**WHAT IS ALREADY KNOWN ON THIS TOPIC**

- Complete blood count (CBC) is widely used in adults but is often dismissed in neonatal diagnostics due to perceived limited utility.
- Current National Institute for Health and Care Excellence guidelines exclude CBC from sepsis evaluation, citing low-quality evidence for the diagnostic values of the white cell count.
- However, these guidelines do not consider the full scope of parameters available from CBC, including novel immune cell activation parameters available from modern analysers.

**WHAT THIS STUDY ADDS**

- This study highlights the diagnostic potential of functional CBC parameters, specifically the immature-to-total neutrophil ratio and mean neutrophil volume, which exhibit higher accuracy than traditional cell counts.
- These findings suggest that these parameters could complement existing diagnostic tools for neonatal sepsis.

**HOW THIS STUDY MIGHT AFFECT RESEARCH, PRACTICE OR POLICY**

- Integrating CBC functional parameters into diagnostic protocols could improve the detection of neonatal sepsis and reduce unnecessary antibiotic usage.
- This study highlights the need for future research on CBC-based diagnostics in neonates and a re-evaluation of the current guidelines.

## Introduction

 Approximately 100000 babies are admitted to neonatal units in the UK each year, primarily due to preterm birth (gestational age <37 weeks) and suspected infections. Neonatal sepsis, a significant cause of morbidity and mortality, accounted for 4640 cases in the UK and 6.31 million cases globally in 2019, as defined by International Classification of Diseases (ICD-10) codes for bacterial infections.^1^

Accurate and timely diagnosis of neonatal sepsis is critical to improving outcomes. Current UK guidelines, published by the National Institute for Health and Care Excellence (NICE) in 2021, recommend blood cultures as the gold standard.^2^ However, blood cultures are often negative in neonatal sepsis, with sensitivities as low as 40%, and results can take up to 72 hours, delaying definitive treatment decisions.^3^ Baseline C reactive protein (CRP) measurements are recommended to monitor treatment efficacy, but their delayed sensitivity limits their role in early diagnosis.^4^

Despite its widespread use outside neonatal care, complete blood count (CBC) is not included in the NICE guidelines for diagnosing sepsis due to insufficient evidence supporting the diagnostic utility of the white cell count (WCC).^5^ In contrast, CBC is a well-established diagnostic tool in adults with suspected sepsis.^6^ Studies in adults have demonstrated significant differences in neutrophil counts between infected and non-infected patients at various time points after diagnosis (p<0.001 at 2 days, p=0.001 at 4 days and p=0.013 at 7 days).^6^

CBC is a rapid, inexpensive test that requires minimal blood volume (as little as 20 µL) and is routinely measured in neonates to monitor haemoglobin levels and detect anaemia.^7^ Recent innovations in automated haematology analysers enable the measurement of functional parameters such as immature-to-total neutrophil ratio (ITR) and mean neutrophil volume (MNV), providing a fresh perspective on the diagnostic utility of CBC. ITR is a measure of neutrophil regeneration—a higher ITR indicates an increase in immature neutrophil production in response to infection.^8^ Immature neutrophils are larger than mature neutrophils, so an increase in MNV also occurs.^8^

This systematic review and meta-analysis aims to evaluate the diagnostic accuracy of CBC parameters, particularly functional markers, for neonatal sepsis. By leveraging recent advances in CBC technology and addressing limitations of prior diagnostic approaches, our study seeks to reframe CBC as a potential adjunctive tool for improving early detection and management of neonatal sepsis.

## Methods

This review follows the Cochrane Library guidelines for conducting a diagnostic accuracy systematic review^9^ and was registered prospectively with PROSPERO (CRD42023476510) on 05 December 2023.

### Eligibility criteria, information sources and search strategy

We conducted an electronic search for studies reporting the diagnostic accuracy of CBC for neonatal sepsis. Eligibility criteria included prospective, retrospective and cross-sectional observational studies conducted on humans and published in English. Case-control studies were included despite their limitations, as they provide valuable insights into diagnostic test performance under specific conditions. Studies with heterogeneous sepsis definitions (eg, non-culture-proven cases) were excluded to ensure diagnostic consistency, thus only studies providing culture-proven sepsis as a case definition were included. Abstracts, case reports and conference proceedings were excluded due to insufficient detail on diagnostic accuracy measures.

Searches were performed in the MEDLINE and Embase via Ovid, Cochrane and CINAHL databases from inception to 28 October 2024. Full search strategies for each database are provided in the online supplemental appendix. To identify additional studies, reference lists of included studies and previously published reviews were manually screened. All search results were imported into Covidence (Melbourne, Australia) for screening.

### Study selection

Two reviewers (EH, MA) independently screened titles and abstracts for relevance, with discrepancies resolved by a third assessor (MV). Full texts of selected studies were reviewed to confirm eligibility. Heterogeneous sepsis definitions were mitigated by excluding studies that did not define cases using culture-proven sepsis. Studies with undefined control groups or inadequate reporting of CBC parameters were also excluded.

### Data extraction

For each study, diagnostic accuracy measures including sensitivity (the proportion of correctly identified septic patients), specificity (the proportion of correctly identified non-septic patients), positive predictive value (PPV; the probability that a positive result is correct) and negative predictive value (NPV; the probability that a negative result is correct) were recorded in an Excel spreadsheet. Where PPV and NPV were not directly reported, they were calculated using sensitivity, specificity and the prevalence of sepsis in each study. True positives, true negatives, false positives and false negatives were also derived. Case definitions for neonatal sepsis (eg, positive culture with or without clinical signs of sepsis) and control definitions were also documented.

### Assessment of applicability

Applicability was assessed using a modified tool derived from the Quality Assessment of Diagnostic Accuracy Studies (QUADAS-2) tool,^10^ the Critical Appraisal Skills Programme diagnostic checklist^11^ and the Newcastle-Ottawa Scale.^12^Selected questions ensured relevance to the research objective. Two reviewers (EH, MA) independently applied the tool, with disagreements resolved by a third reviewer (MV). Studies scoring ≥6/8 were deemed applicable, although all studies were included regardless of score. The use of culture-proven sepsis as a case definition was required for inclusion.

### Data synthesis

All analyses were performed using RStudio (2023.06.1.524, R V.4.3.0). Patient characteristics and biomarker values were summarised as means±SD or medians with IQRs, according to underlying distributions. Optimal thresholds (the threshold at which a test result is considered positive or negative) for each parameter were identified by the highest Youden Index (a measure of overall test performance). Diagnostic accuracy was assessed using a bivariate model (mada::reitsma), incorporating sensitivity, specificity and the area under the curve (AUC; a quantification of overall performance, where values closer to 1 indicate better discrimination between groups). This model was employed to account for study-level differences in diagnostic thresholds. Heterogeneity was quantified using the I^2^ statistic.

A minimum of three studies was required to perform a meta-analysis for each parameter. Subgroup analyses were not conducted to explore variability across study designs and sampling times due to low study numbers. Publication bias was assessed visually through funnel plots.

## Results

### Study selection

In total, 428 studies were identified through database searches (online supplemental figure S1). After title and abstract screening, 259 studies were excluded, leaving 169 for full-text assessment. Ultimately, 24 studies were included in this systematic review: 14 studies from database screening^13^,^26^ and 10 additional studies identified through reference list screening of systematic reviews (n=5)^27^,^31^ and included studies (n=5).^32^,^36^The reasons for exclusion are presented in online supplemental figure S1. Studies with heterogeneous sepsis definitions (eg, those including non-culture-proven sepsis) were excluded to ensure comparability.

### Study characteristics

Among the 24 studies included in this review, 16 were prospective^13^,^1618 19 21^ and 8 were retrospective^17 20 25 27 28 30 31 35^ (table 1). All studies defined neonatal sepsis based on a positive blood culture. Nineteen studies also included clinical signs,^14^,^1921^ and four included additional laboratory indicators.^15^,^1727^ The median prevalence of sepsis across the included studies was 50.0% (IQR: 40.9%–51.7%).

**Table 1:** Characteristics of studies included in this systematic review

No	Study author and year	Setting	Design	Cases					Controls					Applicability Score	Biomarkers
				Definition	No	Sex (M/F)	Age* (days)	GA* (weeks)	Definition	No	Sex (M/F)	Age* (days)	GA* (weeks)		
#1	Abiramalatha *et al* 2016^13^	India	Pro	BC+	38	25/13	4.0	35.5	No CS/LS	360	195/165	1.0	36.5	8.00	WCC, NEUT, ITR, MNV, PLT
#2	Aboud *et al* 2010^14^	Syria	Pro	BC+/CS	25	13/12	8.6	36.4	No CS/LS	22	8/14	9.6	37.3	5.50	WCC
#3	Arcagok and Karabulut 2019^27^	Turkey	Retro	BC+/CS/LS	67	33/34		39.1	No CS/LS	92	43/49		40.1	6.00	ITR, PLR
#4	Buyukeren *et al* 2021^15^	Turkey	Pro	BC+/CS/LS	77	38/39	15.0	34.3	No CS	131	60/71	9.0	34.8	6.00	DNI
#5	Çelik *et al* 2016^16^	Turkey	Pro	BC+/CS/LS	40	26/14	4.5	32.5	No CS	111	70/41	1.0	34.4	7.00	WCC, NEUT, ITR, MNV, MNC, MNS
#6	Celik *et al* 2019^17^	Turkey	Retro	BC+/CS/LS	110	64/46	8.0	28.0	No SUS	87	40/47	7.0	30.0	6.50	DNI
#7	Elawady *et al* 2013^18^	Egypt	Pro	BC+/CS	25	17/8			No CS/LS	25	15/10			5.50	WCC, NEUT, ITR, PLT
#8	Varal and Dogan 2020^31^	Turkey	Retro	BC+/CS	76	40/36	15.0	29.0	No CS	40	22/18	15.0	28.0	6.50	NLR
#9	Varal *et al* 2023^32^	Turkey	Pro	BC+	64	36/28		28.0	No SUS	44	24/20		29.0	7.00	PLT, MPV
#10	Hanaganahalli *et al* 2018^28^	India	Retro	BC+/CS	64	41/23	7.4	39.6	No CS	71	46/25	3.6	39.8	8.00	MPV
#11	Kumar *et al* 2015^19^	India	Pro	BC+/CS	101	58/43			No CS	96	52/44			5.50	WCC
#12	Leibovitch *et al* 2024^20^	Israel	Retro	BC+	63	34/29		31.1	No SUS	63	28/35		31.7	6.00	MPV
#13	Madani *et al* 2019^29^	Iran	Pro	BC+/CS	20	10/10	7.5		No SUS	20	12/8	2.6		5.00	MPV
#14	Mahmoud *et al* 2022^21^	Egypt	Pro	BC+/CS	80		2.3	38.7	No SUS	80		2.0	38.8	7.00	NLR, PLR
#15	Manucha *et al* 2002^33^	India	Pro	BC+	21				No SUS	40				4.00	WCC, NEUT, ITR, PLT
#16	Nesargi *et al* 2020^22^	India	Pro	BC+/CS	84	44/40	7.0	37.0	No CS/LS	160	88/72	3.0	38.0	7.00	MNV, MNC, MNS
#17	Panda *et al* 2021^30^	India	Retro	BC+/CS	41	27/14		34.2	No CS	52	25/27		33.8	6.00	NLR
#18	Panda *et al* 2022^34^	India	Pro	BC+/CS	43	23/40		33.3	No CS	54	24/30		34.2	7.00	PLT, MPV
#19	Schrama *et al* 2008^23^	Netherlands	Pro	BC+/CS	24	8/16		31.0	No CS	55	27/28		30.1	6.00	ITR
#20	Tosson *et al* 2024^24^	Egypt	Pro	BC+/CS	45	25/20	15.0	38.1	No CS/LS	45	25/20	13.0	38.3	6.50	ITR, MPV
#21	Vardar and Ozek 2023^25^	Turkey	Retro	BC+	77	38/39		28.0	No SUS	77	39/38		28.0	5.50	NLR
#22	Yang *et al* 2016^35^	China	Retro	BC+/CS	60	31/29	17.9	34.3	No CS	60	35/25	18.4	37.5	5.00	WCC
#23	Zaki and Sayed 2009^36^	Egypt	Pro	BC+/CS	58	32/26	8.7	39.3	No SUS	30	16/14	8.8	36.9	6.50	WCC, NEUT, ITR, PLT
#24	Zhang *et al* 2021^26^	China	Pro	BC+/CS	74	45/29		38.0	No SUS	50	25/25		38.0	5.50	NLR, PLR

* Mean or median value.

au, arbitrary units; BC+, positive blood culture; CS, clinical signs of sepsis; DNI, Delta Neutrophil Index; fL, femtoliter; GA, gestational age at birth (weeks); ITR, immature-to-total neutrophil ratio; LS, laboratory signs of sepsis; MNC, mean neutrophil conductivity (au); MNS, mean neutrophil scatter (au); MNV, mean neutrophil volume (au); MPV, mean platelet volume (fL); NEUT, neutrophil count (× 109/L); NLR, neutrophil-to-lymphocyte ratio; PLR, platelet-to-lymphocyte ratio; PLT, platelet count (× 109/L); Pro, prospective; Retro, retrospective; SUS, suspicion of sepsis; WCC, white cell count (× 109/L).

Fifteen studies defined the control group as an absence of clinical signs of sepsis,^13^,^1618 19 22^ and six studies included the absence of laboratory markers.^13 14 18 22 24 27^ Nine did not specify the criteria used to define the control group.^17 20 21 25 26 29 32 33 36^

In total, 1377 neonates with sepsis were included across the 24 studies (table 1). The median gestational age at birth was 34.3 weeks (IQR: 31.1–38.0 weeks), and the median age at sampling was 8.0 days (IQR: 7.0–15.0 days). The control group consisted of 1865 neonates, with a median gestational age at birth of 35.6 weeks (IQR: 31.3–38.0 weeks) and a median age at sampling of 7.0 days (IQR: 2.6–9.6 days). No significant differences in gestational age (p=0.705) or age at sampling (p=0.355) were observed between the sepsis and control groups.

### Level of applicability of included studies

**Figure 1:**
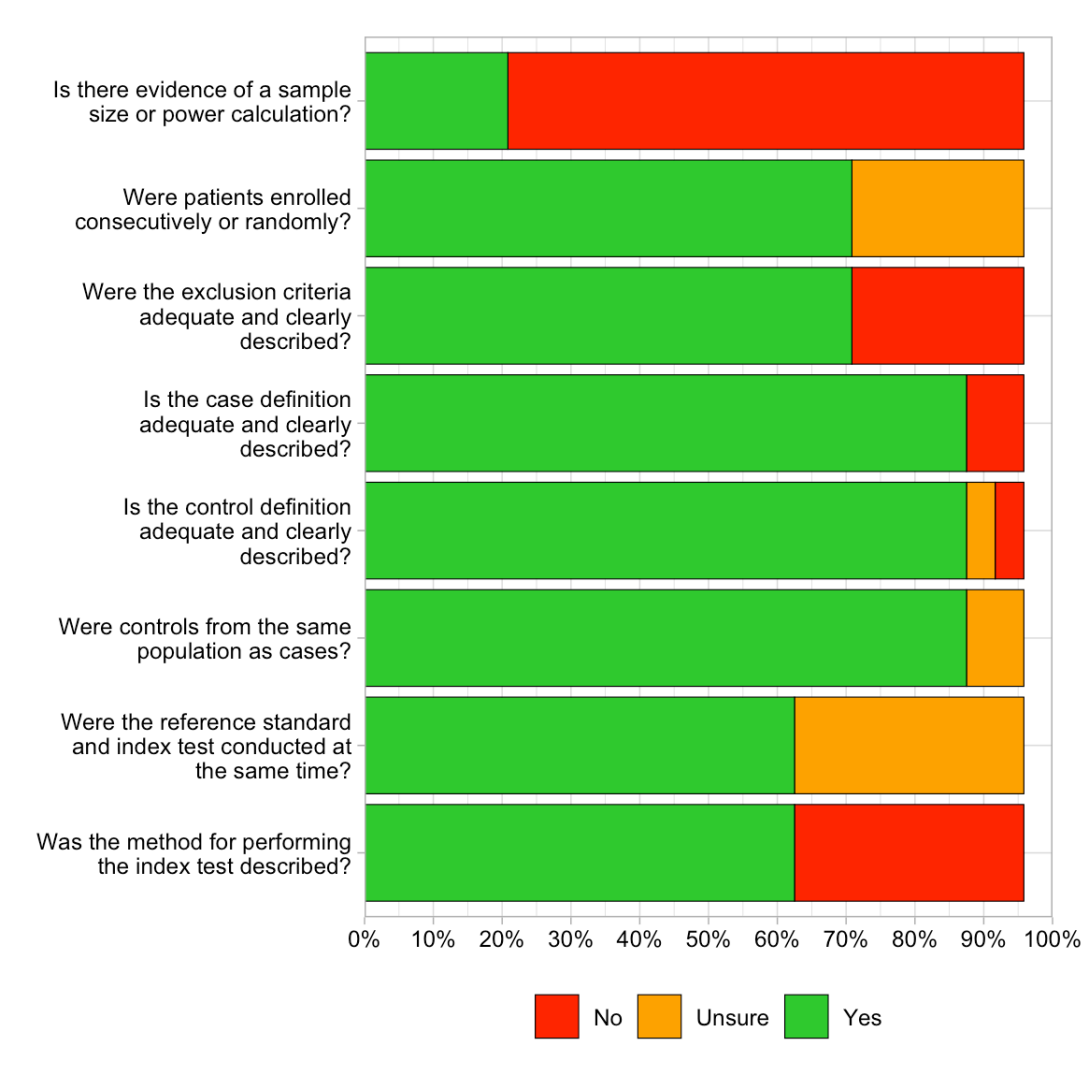
The risk of bias and applicability of the studies included in this systematic review, adapted from the Quality Assessment of Diagnostic Accuracy Studies (QUADAS-2)-2 tool,^10^ the Critical Appraisal Skills Programme diagnostic checklist^11^ and the Newcastle-Ottawa Scale.^12^

Sixteen of the 24 included studies scored 6/8 or higher on the quality assessment tool^1315^,^17 20^ (table 1). Nine studies did not report when samples were taken,^17^,^1921 25 27^ and nine studies did not specify how the CBC was measured.^1419 20 23 25^,^28 35^ Five studies included a sample size or power calculation.^13 21 27 29 32^ While the overall applicability of the included studies was high (figure 1), the lack of detailed reporting in some studies made the assessment of certain aspects of applicability more challenging.

### Synthesis of results

The included studies assessed various CBC parameters: WCC (n=8),^13 14 16 18 19 33 35 36^ neutrophil count (n=5),^13 16 18 33 36^ ITR (n=8),^13 16 18 23 24 27 33 36^ neutrophil-to-lymphocyte ratio (NLR; n=5),^21 25 26 30 31^ Delta Neutrophil Index (DNI; n=2),^15 17^ neutrophil activation markers (MNV (n=3),^13 16 22^ mean neutrophil conductivity (MNC; n=2),^16 22^ mean neutrophil scatter (MNS; n=2),^16 22^ platelet count (n=6),^1318 32^,^34 36^ mean platelet volume (MPV; n=6)^20 24 28 29 32 34^ and platelet-to-lymphocyte ratio (PLR; n=3)^21 26 27^ (online supplemental table S1).

Meta-analyses were conducted for the WCC, neutrophil count, ITR, NLR, MNV, platelet count and MPV (table 2). Due to insufficient studies reporting diagnostic accuracy data, meta-analyses were not performed for DNI, MNC and MNS. Additionally, a reliable meta-analysis could not be conducted for PLR due to substantial heterogeneity in reported values (15.00, 62.40 and 99.57). One study^21^ was excluded from the NLR analysis due to extreme variability compared with other studies (0.80 vs 2.10–4.16), which suggested methodological differences. Therefore, three studies were not included in any meta-analyses.^16 17 21^

**Table 2:** Meta-analysis results for parameters of the complete blood count investigated in this review

Biomarker	Number of studies	Case values*	Control values*	Optimal threshold	Sensitivity*	Specificity*	AUC	I^2^
WCC	8	15.55 (11.19–16.54)	10.89 (9.02–12.76)	> 10.50	55.06% (38.06%–70.95%)	83.33% (73.44%–90.03%)	0.786	0.29
NEUT	5	6.90 (6.90–7.76)	6.51 (4.90–6.51)	> 4.13	67.16% (46.77%–82.64%)	75.93% (63.21%–85.27%)	0.783	<0.01
ITR	8	0.30 (0.18–0.33)	0.10 (0.09–0.11)	> 0.20	66.33% (58.55%–73.31%)	85.41% (74.95%–91.97%)	0.773	<0.01
NLR	4	3.20 (2.10–4.16)	1.40 (0.70–2.34)	> 3.17	69.14% (63.30%–74.42%)	72.63% (54.80%–85.31%)	0.685	0.03
MNV	3	180.30 (161.60–180.30)	146.90 (146.90–150.00)	> 157.90	76.64% (27.51%–96.59%)	90.88% (70.18%–97.69%)	0.925	0.85
PLT	6	187.15 (109.00–220.00)	285.19 (251.00–289.00)	< 278	66.45% (45.69%–82.34%)	81.41% (66.52%–90.62%)	0.815	0.52
MPV	6	9.70 (9.56–9.97)	8.90 (8.88–9.22)	> 11.60	67.72% (58.68%–75.60%)	73.71% (55.82%–86.15%)	0.735	0.39
ITR>0.20	5	0.33 (0.30–0.33)	0.10 (0.09–0.10)	> 0.20	72.46% (60.73%–81.73%)	85.25% (64.44%–94.86%)	0.816	<0.01
PLT<150	4	187.15 (187.15–220.00)	251.00 (251.00–285.19)	< 150	51.24% (38.08%–64.23%)	82.01% (62.16%–92.67%)	0.653	<0.01

* Median (IQR).

AUC, area under the curve; ITR, immature-to-total neutrophil ratio; MNV, mean neutrophil volume (au); MPV, mean platelet volume (fL); NEUT, neutrophil count (× 109/L); NLR, neutrophil-to-lymphocyte ratio; PLT, platelet count (× 109/L); WCC, white cell count (× 109/L).

**Figure 2:**
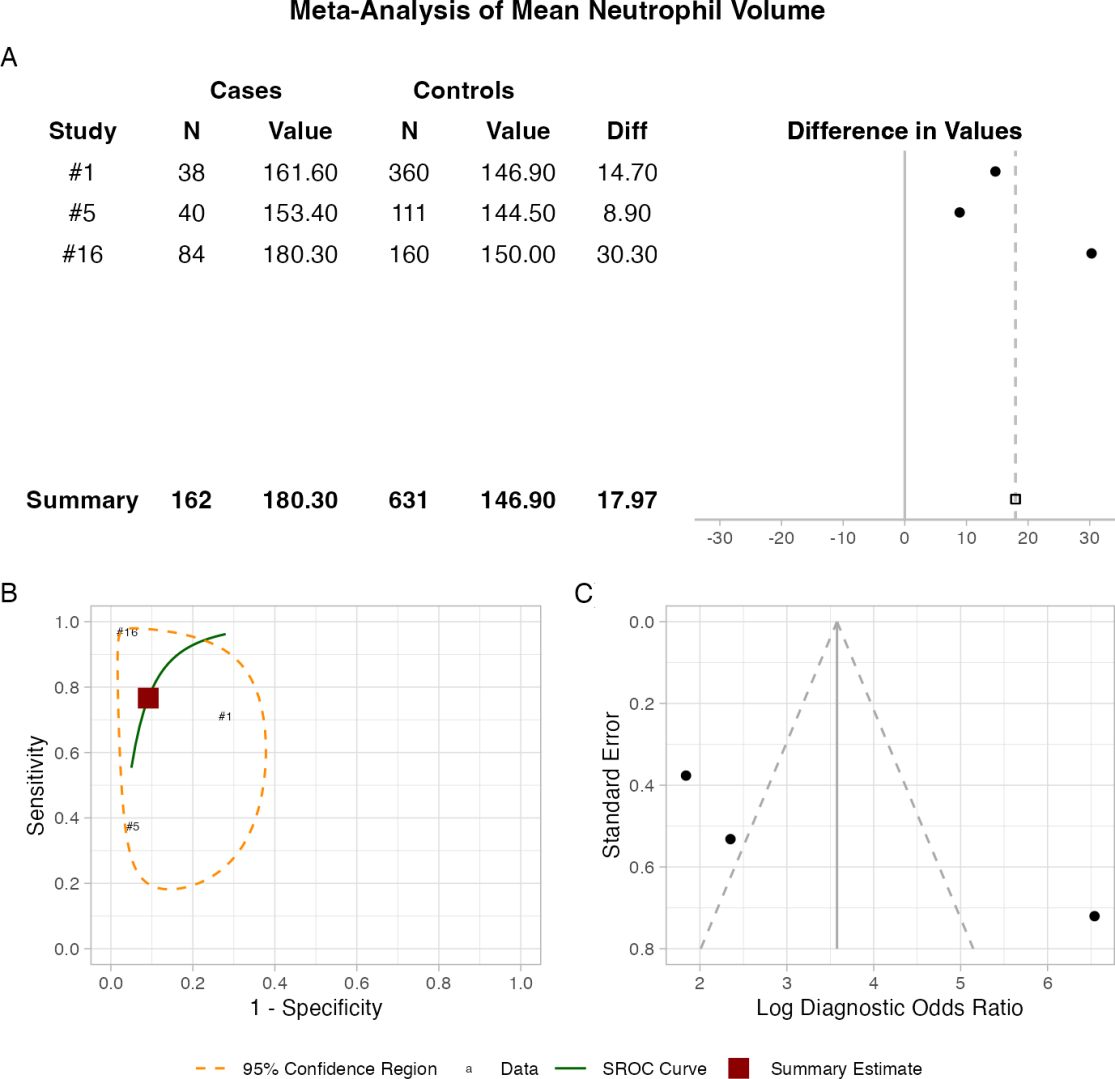
Diagnostic accuracy of mean neutrophil volume (au) for neonatal sepsis. (**A**) Forest plot shows differences in values between cases and controls across individual studies. (**B**) Summary receiver operating characteristic curve (SROC) shows overall diagnostic performance across included studies—individual studies are labelled by their study number. (**C**) Funnel plot provides a visual assessment of publication bias.

MNV showed the highest diagnostic accuracy for neonatal sepsis, with a sensitivity of 76.6%, specificity of 90.9% and an optimal threshold of 157.90 au^22^ (figure 2). Substantial heterogeneity was observed for MNV (I² = 0.85), underscoring the need for standardisation in future research.

**Figure 3:**
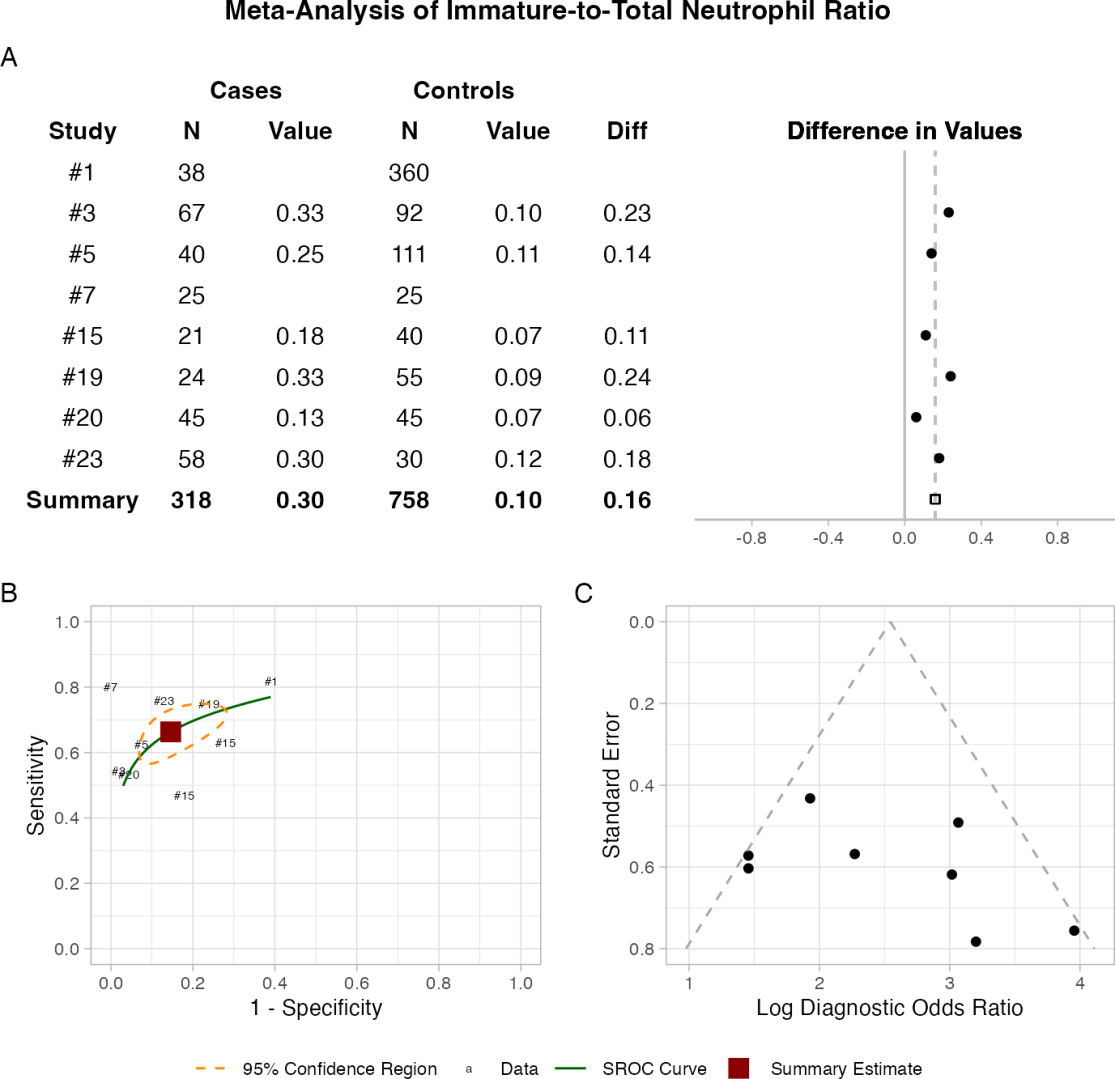
Diagnostic accuracy of immature-to-total neutrophil ratio for neonatal sepsis. (**A**) Forest plot shows differences in values between cases and controls across individual studies. (**B**) Summary receiver operating characteristic (SROC) curve shows overall diagnostic performance across included studies—individual studies are labelled by their study number. (**C**) Funnel plot provides a visual assessment of publication bias.

In contrast, studies reporting on ITR exhibited much less heterogeneity (I^2^<0.10) (figure 3). ITR demonstrated high specificity (85.4%) and moderate sensitivity (66.3%), suggesting its utility as a diagnostic adjunct test. Subgroup analysis of studies reporting an optimal threshold of 0.20 (n=5)^13 18 23 27 36^ revealed a higher pooled sensitivity (72.5%) compared with the full cohort (66.3%) while specificity remained similar (85.3% vs 85.4%) and the AUC was higher (0.816 vs 0.773) (online supplemental figure S2).

Platelet count demonstrated moderate diagnostic accuracy, with a sensitivity of 66.5%, specificity of 81.4% and an optimum threshold of 278 × 10^9^/L^32^ (online supplemental figure S3). Subgroup analysis of studies reporting an optimal threshold of 150 × 10^9^/L reduced the I^2^ statistic (< 0.10) and resulted in slightly lower diagnostic accuracy (sensitivity: 51.2%; specificity: 82.0%) (online supplemental figure 4).

NLR had the lowest diagnostic accuracy, with a sensitivity of 69.1%, specificity of 72.6% and AUC of 0.685, at an optimal threshold of 3.17^26^ (online supplemental figure S5). The results of meta-analyses for WCC (online supplemental figure S6), neutrophil count (online supplemental figure S7), and MPV (online supplemental figure S8) are presented in table 2. Subgroup analyses based on study design or onset of sepsis could not be conducted due to the low number of studies available for each parameter.

## Discussion

### Principal findings

This systematic review and meta-analysis evaluated the diagnostic utility of CBC parameters for neonatal sepsis, with a particular focus on functional markers. Although ITR and MNC showed moderate diagnostic accuracy, they are insufficient as stand-alone markers. ITR achieved a sensitivity of 66.3% and specificity of 85.4%, with minimal heterogeneity (I² < 0.10), suggesting consistent diagnostic performance across studies. However, its moderate sensitivity means it would miss a significant proportion of true sepsis cases.

MNV demonstrated promising utility, with a pooled sensitivity of 76.6% and specificity of 90.9%. However, the heterogeneity of included studies (I² = 0.85) raises concerns about generalisability. Differences in study design, diagnostic thresholds and analytical techniques likely contribute to this variability. Traditional markers including the WCC displayed lower diagnostic accuracy, reflecting their susceptibility to physiological factors such as perinatal stress, neonatal colonisation and steroid exposure.^37^

### Comparison with existing literature

Our study builds on and differentiates itself from prior systematic reviews. Earlier reviews primarily focused on traditional CBC parameters such as the WCC and platelet count, which were found to have limited diagnostic accuracy for neonatal sepsis.^38 39^ Fowlie *et al* identified a high variability in the WCC, which could be due to factors like perinatal stress and postnatal physiological changes, or due to variability in the methodology of included papers, including case definitions or timing of measurements. Rees *et al* highlighted the need for novel biomarkers, particularly in resource-constrained settings where the cost and availability of tests, especially PCT, pose challenges.

In contrast, this review emphasises functional parameters such as ITR and MNV. Previous reviews have concluded that both the MNV and ITR have high diagnostic accuracy for neonatal sepsis.^40 41^ However, Mishra *et al*^40^ included studies evaluating culture-proven and suspected sepsis, while Zhang *et al*^41^ evaluated studies that defined the control group as neonates with suspected sepsis. These reviews highlight the heterogeneity of definitions within the literature. In contrast, the current study excluded all studies with overlapping case and control definitions to ensure a robust evaluation of the neonatal CBC.

The current gold standard for diagnosing sepsis in neonates, as defined by the NICE guidelines, is a positive blood culture.^2^ However, up to 60% of true sepsis cases are culture-negative, becoming positive only in severe cases of sepsis. Serial CRP measurements are recommended to monitor the progression of sepsis, although CRP levels only begin to rise 12 hours after infection onset, making it unsuitable for early diagnosis.^4^ Moreover, the accuracy of CRP varies significantly, with reported sensitivities ranging from 30% to 80% at symptom onset.^4^

The guidelines also recommend starting antibiotics while awaiting laboratory results.^2^ However, the inappropriate use of antibiotics, especially in culture-negative cases, can have both short-term and long-term health consequences for neonates. For example, a 7-day course of antibiotics increases the risk of necrotising enterocolitis twofold,^42^ and long-term antibiotic exposure has been linked to higher risks of inflammatory bowel disease (OR: 6.34).^43^

The CBC’s affordability, rapid processing and minimal blood volume requirements make it a practical adjunctive tool, even in low-resource settings where advanced technology is becoming more readily available. Functional parameters such as ITR and MNV could enhance early detection and guide antibiotic stewardship, especially when existing tools such as CRP and PCT yield inconclusive or conflicting results.

One application of the CBC, which has been previously investigated by both Narasimha and Kumar^44^ and Huang *et al*,^45^ is the development of diagnostic algorithms incorporating the CBC parameters. Narasimha and Kumar created a scoring system for the CBC whereby all neonates with culture-positive sepsis scored at least 5 out of 10. Similarly, Huang *et al* combined CBC parameters with clinical variables using machine learning. Their highest-performing models achieved specificities greater than 70% with sensitivities of 40%–60%. Since these studies used the CBC in different ways, it is not possible to compare them directly. However, they provide good evidence for investigating the use of the CBC in more sophisticated diagnostic models.

By focusing on functional CBC parameters and leveraging advancements in analyser technology, our study reframes the CBC as more than a traditional diagnostic tool. While its stand-alone utility is limited, integrating CBC parameters with other diagnostic approaches offers a promising pathway for improving neonatal sepsis outcomes. Future research should aim to standardise the use of these parameters, explore their role in combined diagnostic algorithms and validate their performance in prospective clinical settings.

### Strengths and limitations

Decision-making in neonatal sepsis is a critical challenge for neonatologists. A strong evidence base is essential, given the vulnerability of this patient cohort due to their immature immune systems. A key challenge is the lack of a standard definition for neonatal sepsis, with many studies relying solely on clinical signs. However, signs such as tachycardia, tachypnoea and jaundice are non-specific and commonly present in healthy neonates. Our review highlights the challenge of addressing interstudy variability in diagnostic accuracy studies. To enhance the accuracy of our findings, we excluded all studies that did not include a positive culture as part of the sepsis diagnosis, accounting for 42 excluded studies.

The breadth of definitions for control groups in the literature also posed a significant challenge. Thirty-two studies were excluded because their control groups were inadequately defined, often including neonates with clinical signs of sepsis but negative blood cultures. This highlights the overlap between case and control groups in neonatal sepsis research. By excluding these studies, we believe our study provides the most accurate picture of the CBC’s diagnostic utility for neonatal sepsis to date. However, even within the included studies, it is likely that misclassification occurred due to culture-negative septicaemia and blood culture contaminants. The lack of a gold standard sepsis definition continues to weaken the literature on this subject.

Finally, a substantial limitation of this review is the insufficient number of relevant studies available for analysis. There is a clear need for further research on all the parameters evaluated to confirm our findings.

## Conclusion

Building on earlier reviews, our systematic review and meta-analysis evaluates the diagnostic accuracy of the CBC for neonatal sepsis, providing novel insights into their diagnostic potential. The CBC, particularly functional parameters such as ITR and MNV, has potential as a low-cost adjunctive tool in neonatal sepsis diagnosis. However, its moderate diagnostic accuracy underscores the need for further validation and integration with established biomarkers.

## Supplementary material

10.1136/archdischild-2025-328523 online supplemental file 1

10.1136/archdischild-2025-328523 online supplemental figure 1

10.1136/archdischild-2025-328523 online supplemental figure 2

10.1136/archdischild-2025-328523 online supplemental figure 3

10.1136/archdischild-2025-328523 online supplemental figure 4

10.1136/archdischild-2025-328523 online supplemental figure 5

10.1136/archdischild-2025-328523 online supplemental figure 6

10.1136/archdischild-2025-328523 online supplemental figure 7

10.1136/archdischild-2025-328523 online supplemental figure 8

10.1136/archdischild-2025-328523 online supplemental table 1

## Data Availability

All data relevant to the study are included in the article or uploaded as supplementary information.
