# Supplementary material for: Role of complete blood count in the diagnosis of culture-proven neonatal sepsis: a systematic review and meta-analysis
Source: Arch Dis Child. 2025 May 24;110(10):e328523. doi: 10.1136/archdischild-2025-328523 (PMC12505116; doi:10.1136/archdischild-2025-328523)
Supplement: online supplemental file 1 [file archdischild-110-10-s001.pdf]

Appendix

Search Terms

	Medline and Embase	CINAHL	Cochrane
1	Exp Neonatal Sepsis/	(MH “Neonatal Sepsis”)	MeSH descriptor: [Neonatal Sepsis] explode all trees
2	(Neonatal adj2 infection).mp.	Neonatal infection	(Neonatal infection)
3	1 OR 2	1 OR 2	1 OR 2
4	Exp Leukocytes/	(MH “Leukocytes”)	MeSH descriptor: [Leukocytes] explode all trees
5	Exp Blood cell count/	(MH “Blood cell count”)	MeSH descriptor: [Blood cell count] explode all trees
6	Exp Neutrophils/	(MH “Neutrophils”)	MeSH descriptor: [Neutrophils] explode all trees
7	Exp Lymphocytes/	(MH “Lymphocytes”)	MeSH descriptor: [Lymphocytes] explode all trees
8	Exp Basophils/	(MH “Basophils”)	MeSH descriptor: [Basophils] explode all trees
9	Exp Monocytes/	(MH “Monocytes”)	MeSH descriptor: [Monocytes] explode all trees
10	Exp Macrophages/	(MH “Macrophages”)	MeSH descriptor: [Macrophages] explode all trees
11	Exp Eosinophils/	(MH “Eosinophils”)	MeSH descriptor: [Eosinophils] explode all trees
12	4 OR 5 OR 6 OR 7 OR 8 OR 9 OR 10 OR 11	4 OR 5 OR 6 OR 7 OR 8 OR 9 OR 10 OR 11	4 OR 5 OR 6 OR 7 OR 8 OR 9 OR 10 OR 11
13	Exp “Sensitivity and Specificity”/	(MH “Sensitivity and Specificity”)	MeSH descriptor: [Sensitivity and Specificity] explode all trees
14	(Sensitivity).mp.	Sensitivity	(Sensitivity)
15	(Specificity).mp.	Specificity	(Specificity)
16	(Accuracy).mp.	Accuracy	(Accuracy)
17	13 OR 14 OR 15 OR 16	13 OR 14 OR 15 OR 16	13 OR 14 OR 15 OR 16
18	3 AND 12 AND 17	3 AND 12 AND 17	3 AND 12 AND 17
