# Supplementary figures and images for: Role of complete blood count in the diagnosis of culture-proven neonatal sepsis: a systematic review and meta-analysis
Source: Arch Dis Child. 2025 May 24;110(10):e328523. doi: 10.1136/archdischild-2025-328523 (PMC12505116; doi:10.1136/archdischild-2025-328523)

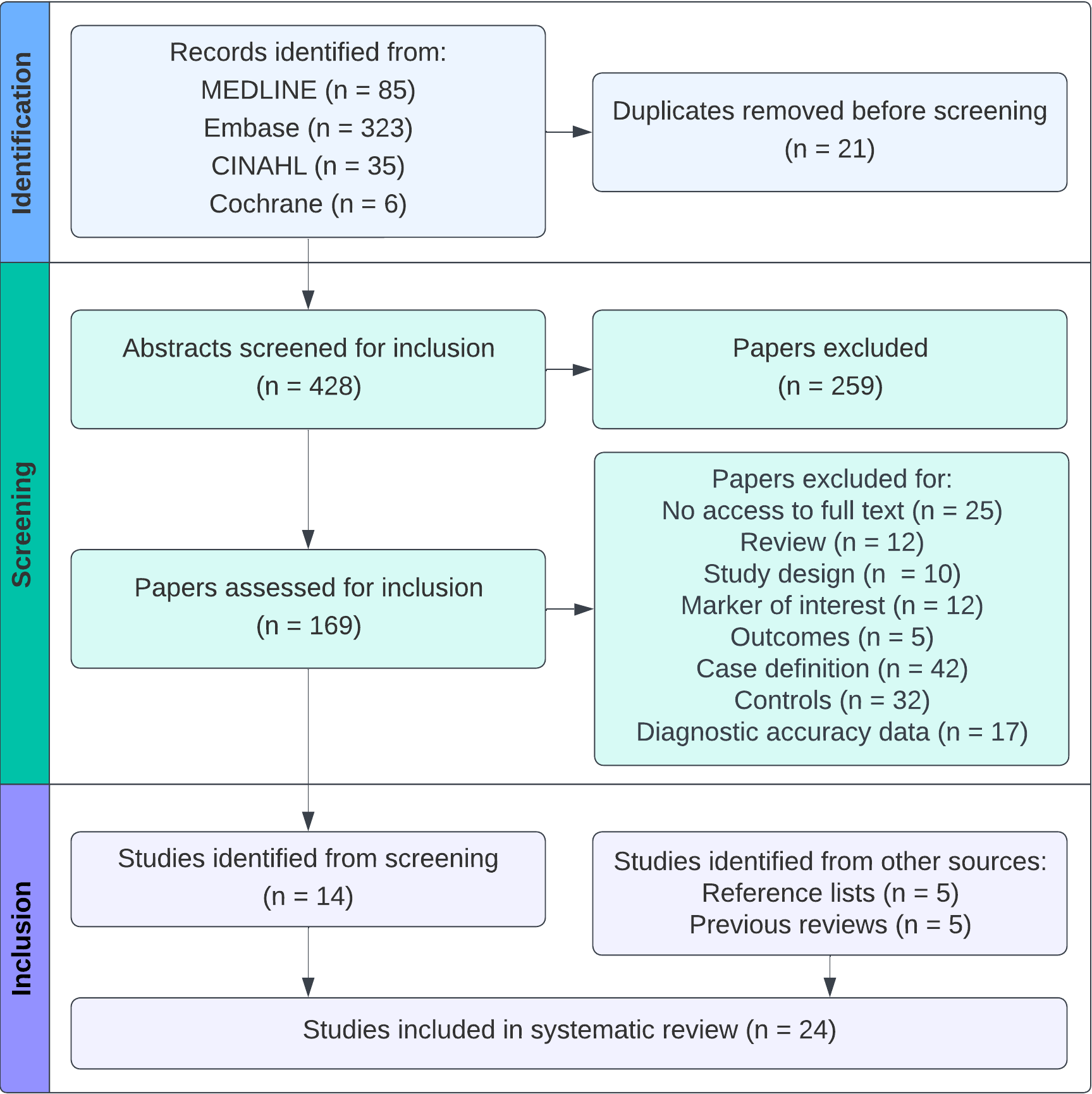

Supplement: online supplemental figure 1 [file archdischild-110-10-s002.png]

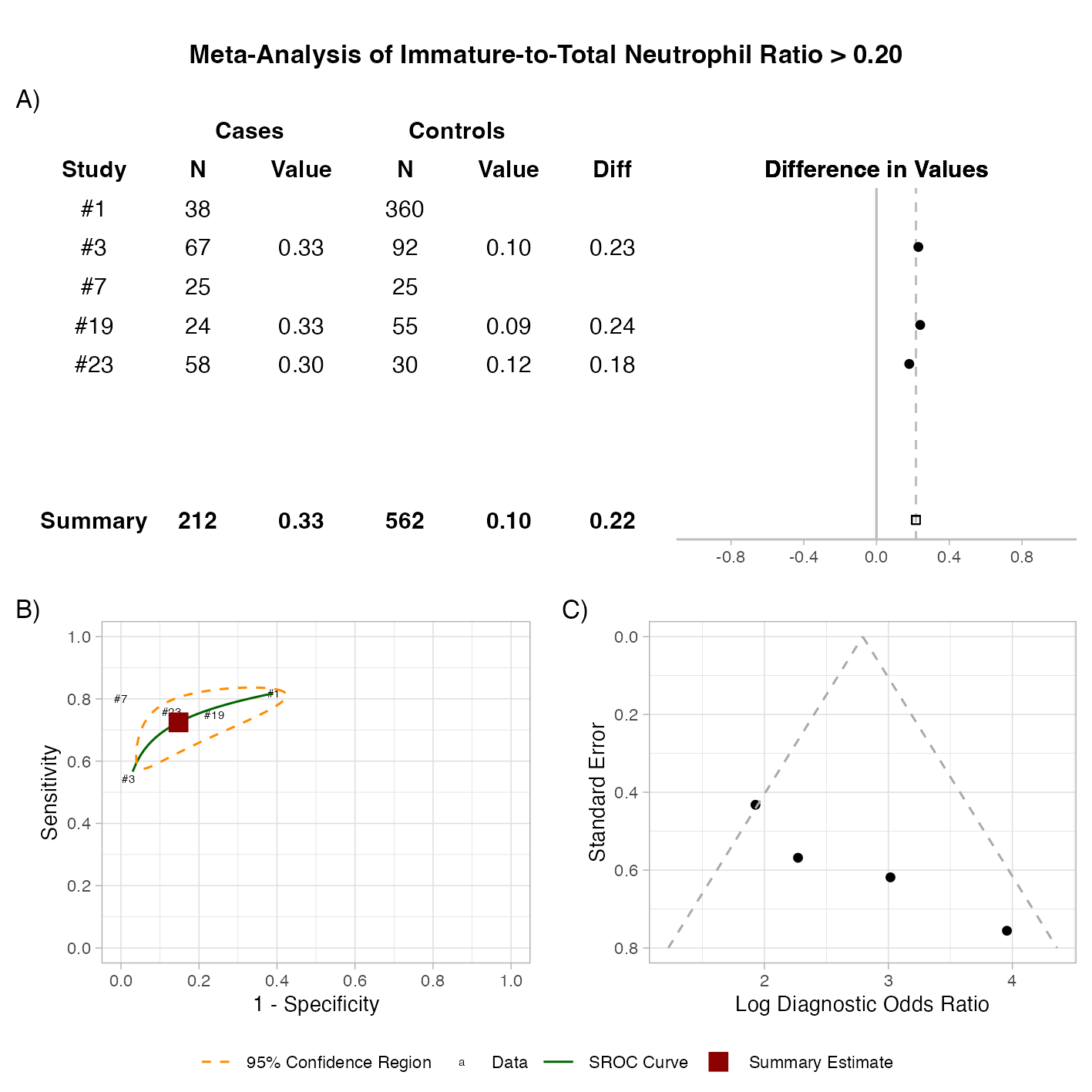

Supplement: online supplemental figure 2 [file archdischild-110-10-s003.png]

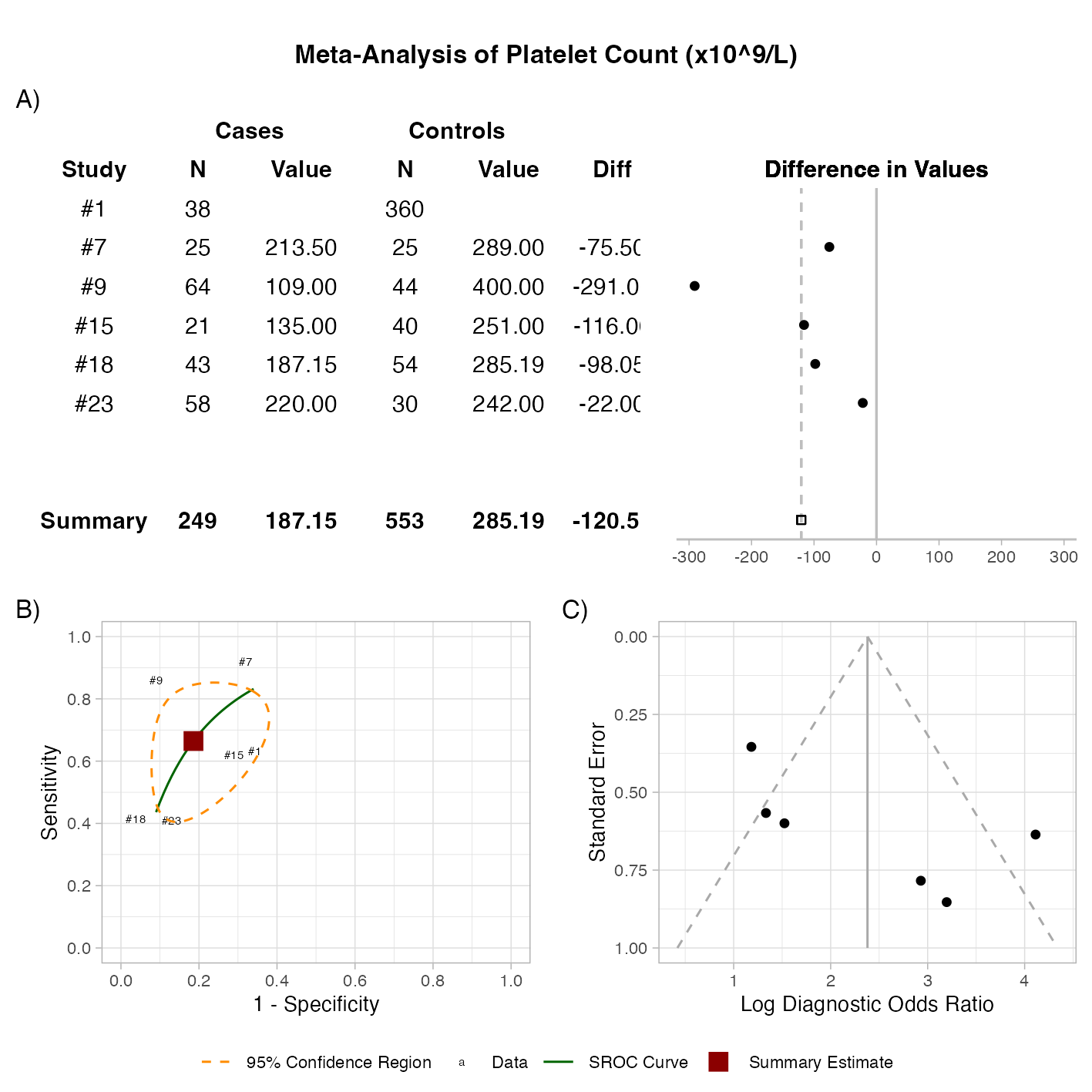

Supplement: online supplemental figure 3 [file archdischild-110-10-s004.png]

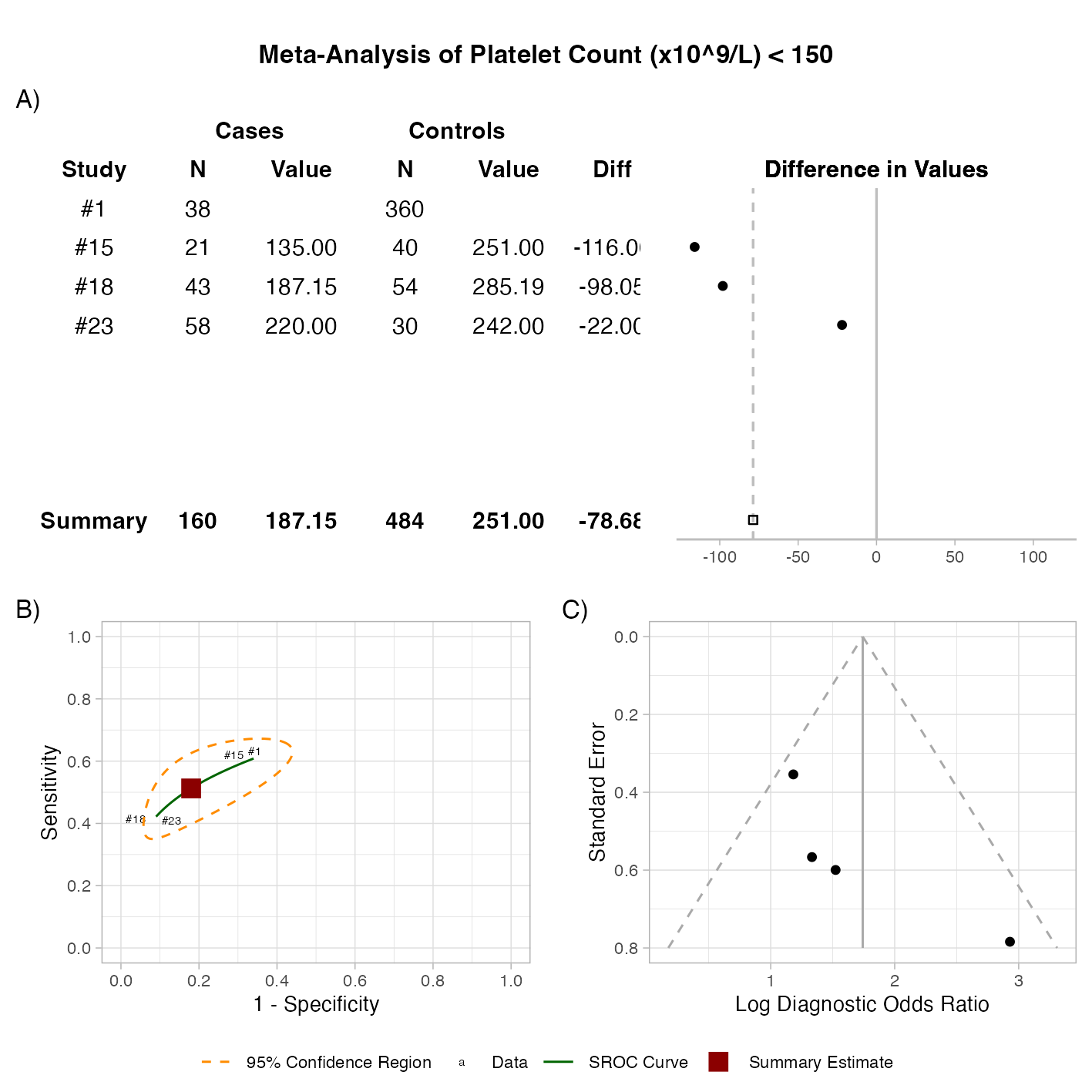

Supplement: online supplemental figure 4 [file archdischild-110-10-s005.png]

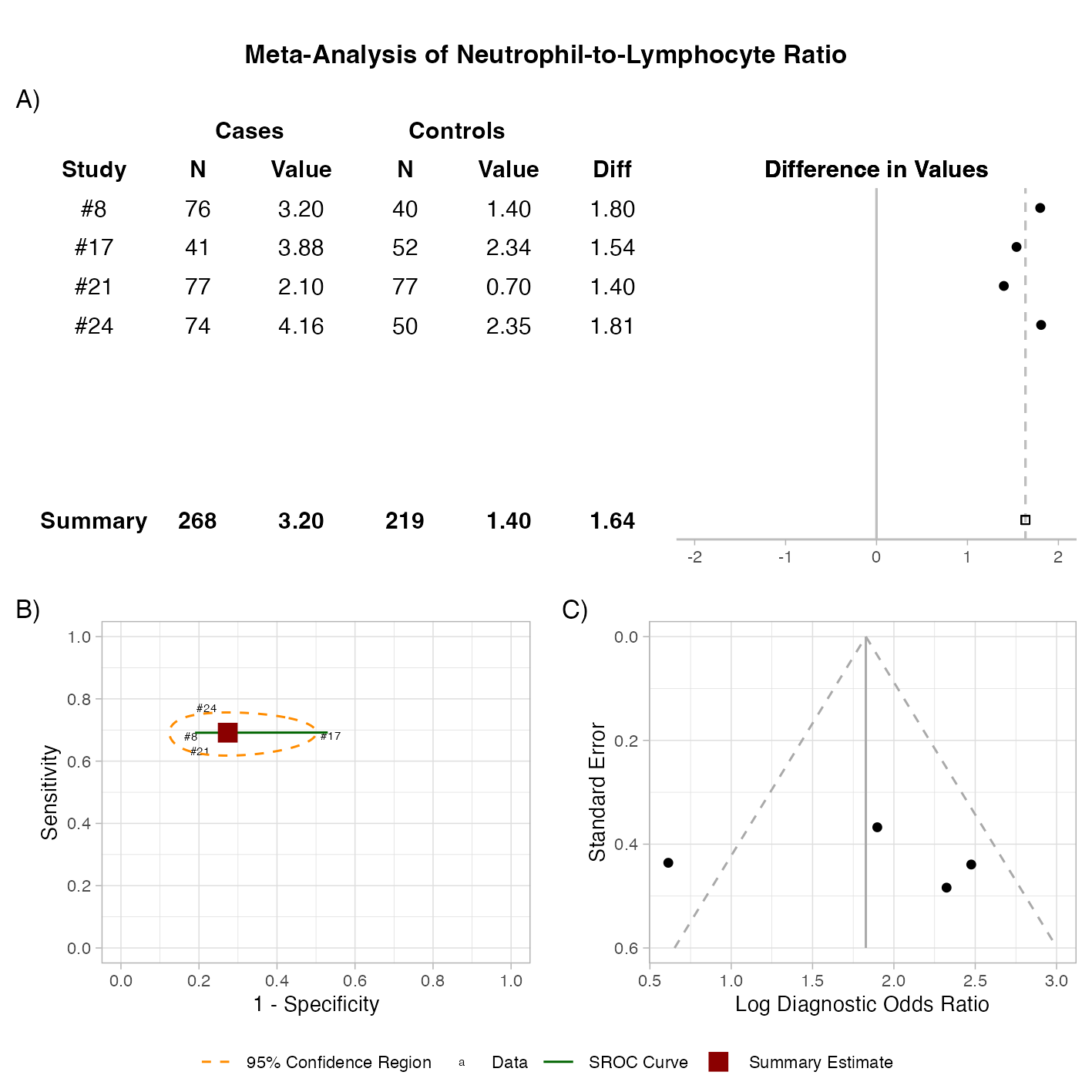

Supplement: online supplemental figure 5 [file archdischild-110-10-s006.png]

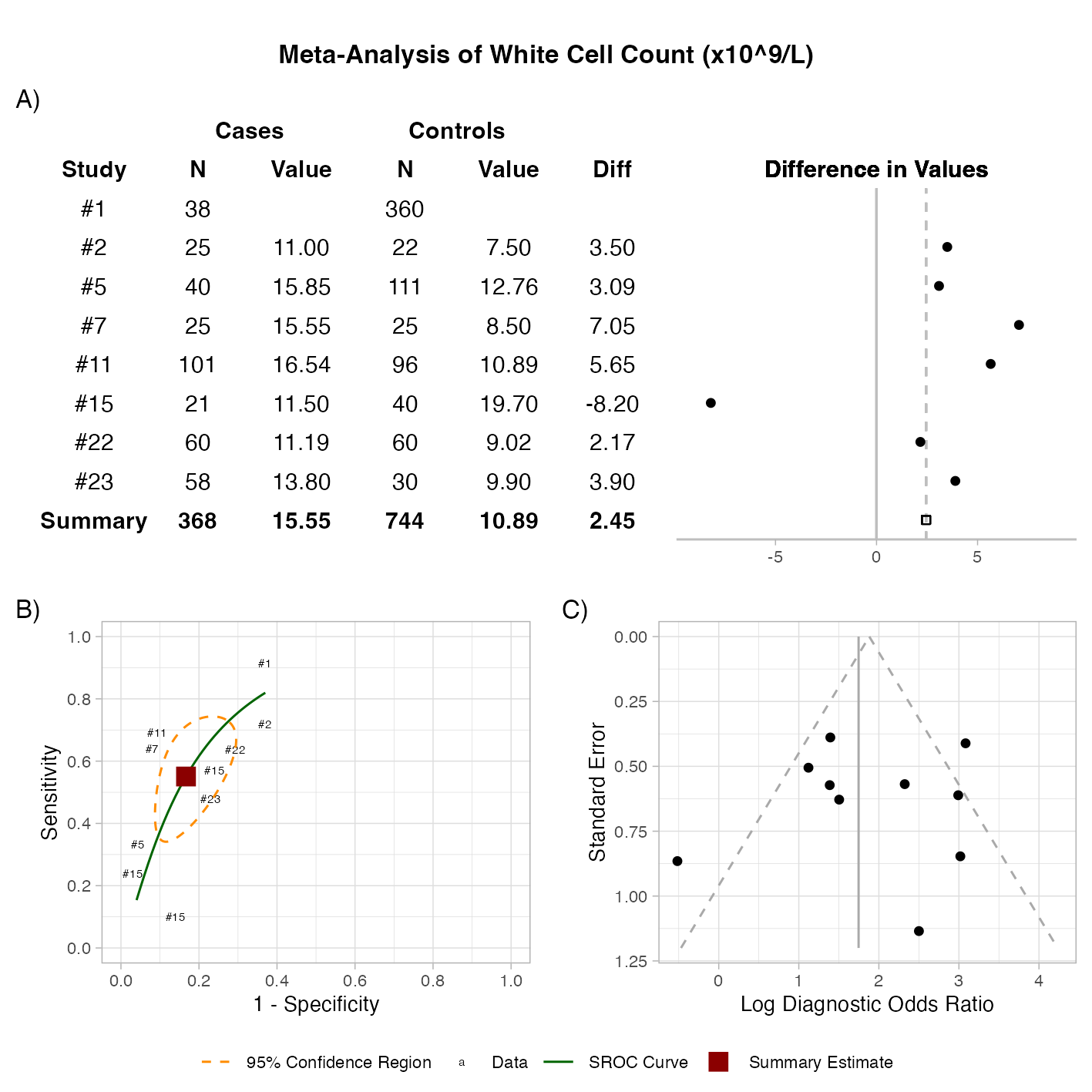

Supplement: online supplemental figure 6 [file archdischild-110-10-s007.png]

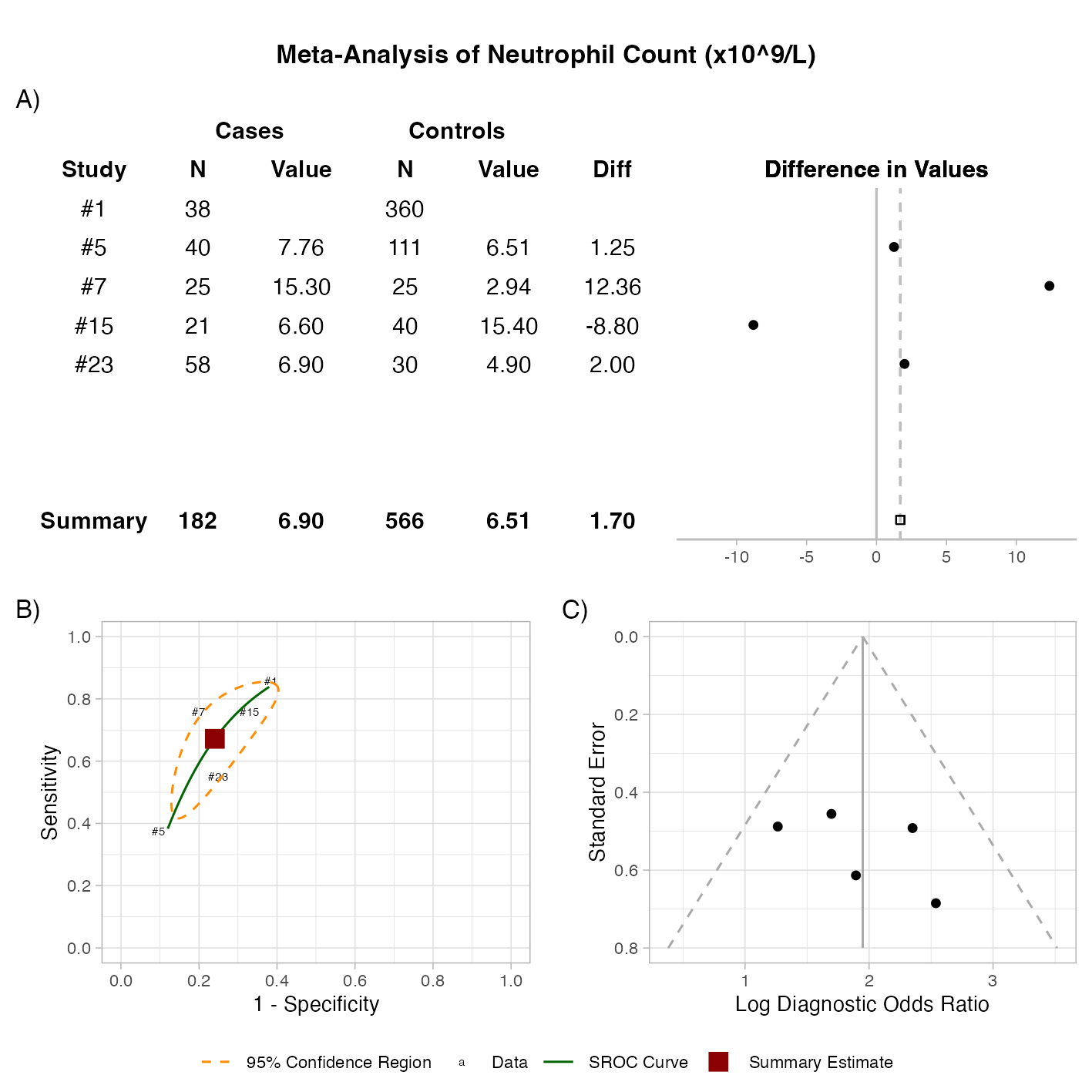

Supplement: online supplemental figure 7 [file archdischild-110-10-s008.png]

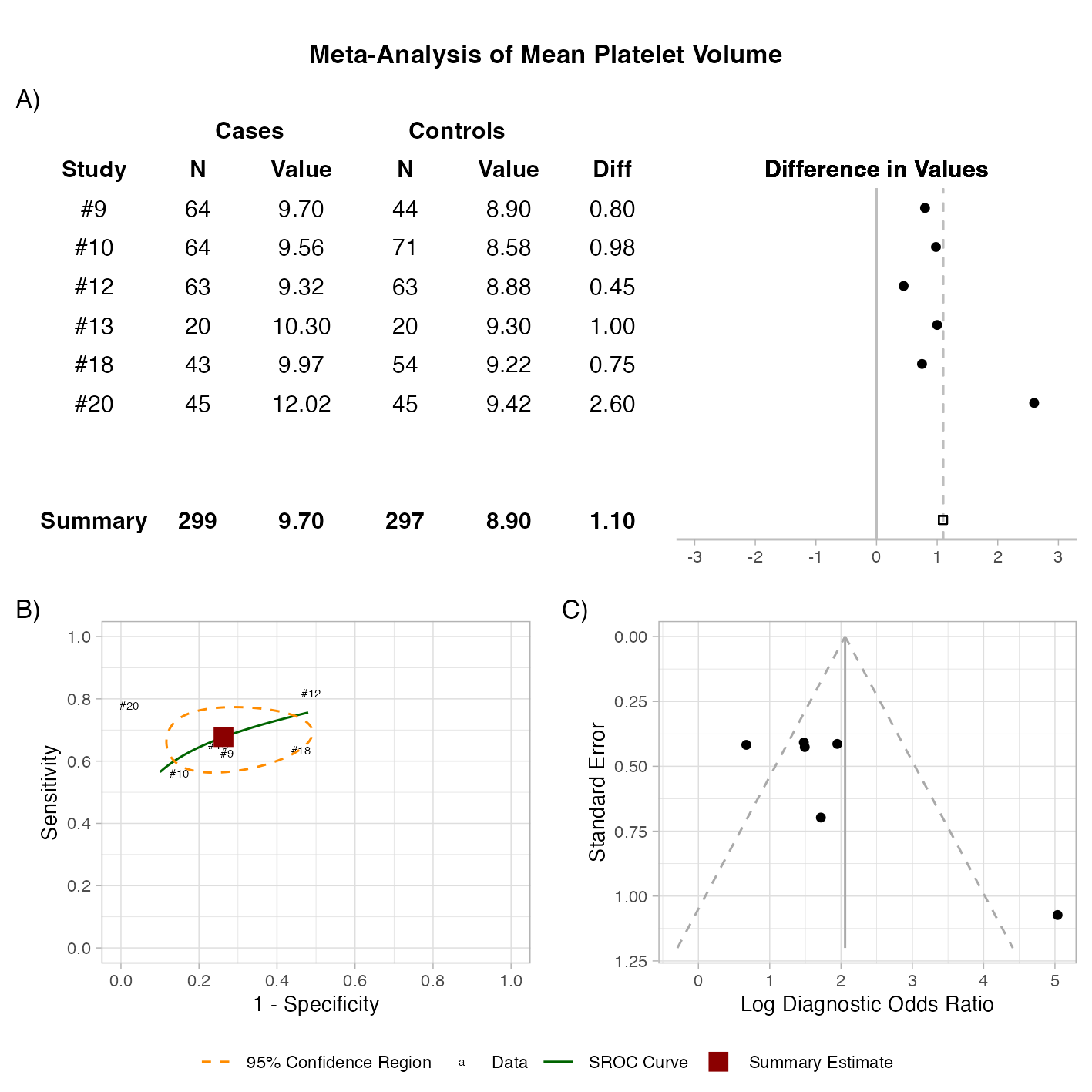

Supplement: online supplemental figure 8 [file archdischild-110-10-s009.png]
