# Supplementary material for: Role of complete blood count in the diagnosis of culture-proven neonatal sepsis: a systematic review and meta-analysis
Source: Arch Dis Child. 2025 May 24;110(10):e328523. doi: 10.1136/archdischild-2025-328523 (PMC12505116; doi:10.1136/archdischild-2025-328523)
Supplement: online supplemental table 1 [file archdischild-110-10-s010.pdf]

Supplementary Tables

Table S1: Individual parameter values and diagnostic accuracy data from studies included in this review. Case and control values are presented as (average ± standard deviation (minimum-maximum)). Abbreviations: PPV - positive predictive value; NPV - negative predictive value; LR+ - positive likelihood ratio; LR- - negative likelihood ratio; TP - true positive; TN - true negative; FP - false positive; FN - false negative; AUC - area under the receiver operator curve. .[a]mean or median (minimum – maximum); [b]excluded from meta-analysis; [c]optimal threshold, based on Youden index.

Study No	Case Values[a]	Control Values[a]	Threshold	Sensitivity	Specificity	PPV	NPV	LR^+	LR^-	TP	TN	FP	FN	AUC	Youden
White cell count (x10^9/L)															
#1			< 5.00	91.40%	63.00%	20.83%	98.70%	2.47	0.14	35	227	133	3		0.544
#11	16.54<br>(0.95 - 33.22)	10.89<br>(3.58 - 22.08)		69.30%	90.70%	88.61%	73.73%	7.45	0.34	70	87	9	31		0.600
#15	11.50 ± 7.00	19.70 ± 5.80	< 5.00	24.00%	97.00%	83.33%	70.91%	8.00	0.78	5	39	1	16		0.210
#15			< 10.00	57.00%	76.00%	54.55%	76.92%	2.38	0.57	12	30	10	9		0.330
#15			> 20.00	10.00%	86.00%	25.00%	64.15%	0.71	1.05	2	34	6	19		-0.040
#2	11.00 ± 6.50<br>(1.80 - 27.80)	7.50 ± 2.80<br>(3.70 - 14.00)	< 4.00 or<br>> 12.00	72.00%	63.00%	69.23%	66.67%	1.95	0.44	18	14	8	7	0.670	0.350
#22	11.19 ± 4.94<br>(9.69 - 12.70)	9.02 ± 3.04<br>(8.05 - 9.97)	> 8.99	63.64%	70.73%	67.86%	65.63%	2.17	0.51	38	42	18	22	0.671	0.344
#23	13.80 ± 9.40	9.90 ± 4.50	< 5.00 or<br>> 20.00	48.00%	77.00%	80.00%	43.40%	2.09	0.68	28	23	7	30		0.250

Study No	Case Values[a]	Control Values[a]	Threshold	Sensitivity	Specificity	PPV	NPV	LR+	LR-	TP	TN	FP	FN	AUC	Youden
#5	15.85 ± 13.08	12.76 ± 10.22	> 19.60	33.30%	95.70%	72.22%	79.70%	7.74	0.70	13	106	5	27	0.590	0.290
#7	15.55 (3.50 - 27.60)	8.50 (5.70 - 11.30)	> 10.50[c]	64.00%	92.00%	88.89%	71.88%	8.00	0.39	16	23	2	9	0.710	0.560
Neutrophil count (x10^9/L)															
#1			< 1.50	85.70%	61.50%	19.19%	97.79%	2.23	0.23	33	221	139	5		0.472
#15	6.60 ± 5.10	15.40 ± 5.50	< 8.00	76.00%	67.00%	55.17%	84.38%	2.30	0.36	16	27	13	5		0.430
#23	6.90 ± 4.20	4.90 ± 1.90	< 2.00 or > 7.50	55.00%	75.00%	80.00%	46.94%	2.20	0.60	32	23	8	26		0.300
#5	7.76 ± 6.37	6.51 ± 4.42	> 10.08	37.50%	90.30%	57.69%	80.00%	3.87	0.69	15	100	11	25	0.542	0.278
#7	15.30 (1.48 - 29.12)	2.94 (1.70 - 4.18)	> 4.13[c]	76.00%	80.00%	79.17%	76.92%	3.80	0.30	19	20	5	6	0.816	0.560
Immature to total neutrophil ratio															
#1			> 0.20	81.80%	60.80%	18.02%	96.90%	2.09	0.30	31	219	141	7		0.426
#15	0.18 ± 0.09	0.07 ± 0.12	> 0.14	63.00%	72.00%	54.17%	78.38%	2.25	0.51	13	29	11	8		0.350
#15			> 0.16	47.00%	82.00%	58.82%	75.00%	2.61	0.65	10	33	7	11		0.290

Study No	Case Values[a]	Control Values[a]	Threshold	Sensitivity	Specificity	PPV	NPV	LR^+	LR^-	TP	TN	FP	FN	AUC	Youden
#19	0.33 (0.07 - 0.84)	0.09 (0.00 - 0.40)	> 0.20	75.00%	76.00%	58.06%	87.50%	3.13	0.33	18	42	13	6	0.903	0.510
#20	0.13 (0.03 - 0.32)	0.07 (0.00 - 0.14)	> 0.13	53.30%	95.60%	92.31%	67.19%	12.11	0.49	24	43	2	21	0.780	0.489
#23	0.30 ± 0.17	0.12 ± 0.12	> 0.20	76.00%	87.00%	91.67%	65.00%	5.85	0.28	44	26	4	14	0.800	0.630
#3	0.33 ± 0.08	0.10 ± 0.05	> 0.22	54.30%	98.10%	94.74%	74.38%	28.58	0.47	36	90	2	31	0.955	0.524
#5	0.25 ± 0.14	0.11 ± 0.09	> 0.19	62.50%	92.50%	75.76%	87.29%	8.33	0.41	25	103	8	15	0.815	0.550
#7	(0.02 - 0.58)	(0.02 - 0.10)	> 0.20[c]	80.00%	100.00%	100.00%	83.33%	Inf	0.20	20	25	0	5	0.902	0.800
Neutrophil to lymphocyte ratio															
#14[b]	0.80 ± 1.10 (0.00 - 1.10)	0.08 ± 0.30 (0.00 - 0.00)	> 0.10	67.00%	99.00%	98.18%	75.24%	67.00	0.33	54	79	1	26	0.790	0.660
#17	3.88 ± 1.78	2.34 ± 1.98	> 1.70	68.30%	46.20%	50.00%	64.86%	1.27	0.69	28	24	28	13	0.623	0.145
#21	2.10 (0.80 - 5.50)	0.70 (0.50 - 1.27)	> 1.37	63.30%	79.70%	75.38%	68.54%	3.12	0.46	49	61	16	28	0.764	0.430
#24	4.16 (3.26 - 5.61)	2.35 (1.97 - 3.16)	> 3.17[c]	77.00%	78.00%	83.82%	69.64%	3.50	0.29	57	39	11	17	0.788	0.550

Study No	Case Values[a]	Control Values[a]	Threshold	Sensitivity	Specificity	PPV	NPV	LR^+	LR^-	TP	TN	FP	FN	AUC	Youden
#8	3.20 ± 2.50	1.40 ± 0.20	> 1.57	68.00%	82.00%	88.14%	57.89%	3.78	0.39	52	33	7	24	0.723	0.500
Delta neutrophil index															
#4	1.50 (1.00 - 2.50)	0.10 (0.00 - 0.30)	> 0.65	96.20%	97.40%	96.10%	97.71%	37.00	0.04	74	128	3	3		0.936
#6	20.80 (0.10 - 65.80)	1.40 (0.10 - 16.50)	> 4.60	85.00%	80.00%	84.68%	80.46%	4.25	0.19	94	70	17	17	0.880	0.650
Mean neutrophil volume (au)															
#1	161.60	146.90	> 151	71.30%	71.90%	21.09%	95.93%	2.54	0.40	27	259	101	11	0.783	0.432
#16	180.30 (169.20 - 194.20)	150.00 (146.30 - 153.50)	> 157.90[b]	97.00%	96.00%	93.10%	98.09%	24.25	0.03	81	154	6	3	0.990	0.930
#5	153.40 ± 15.90	144.50 ± 10.20	> 159.50	37.50%	94.60%	71.43%	80.77%	6.94	0.66	15	105	6	25	0.634	0.321
Mean neutrophil conductivity (au)															
#16	141.70 (132.70 - 150.30)	137.20 (134.10 - 144.70)	> 137.90	63.00%	57.00%	43.44%	74.59%	1.47	0.65	53	91	69	31	0.590	0.200
#5	138.00 ± 8.90	142.30 ± 7.80	< 144.50	78.40%	46.80%	34.44%	85.25%	1.47	0.46	31	52	59	9	0.626	0.252
Mean neutrophil scatter (au)															
#16	119.60 (117.30 - 122.80)	125.70 (119.80 - 129.70)	< 120.30	47.00%	32.00%	26.35%	53.13%	0.69	1.66	39	51	109	45	0.280	-0.210

Study No	Case Values[a]	Control Values[a]	Threshold	Sensitivity	Specificity	PPV	NPV	LR^+	LR^-	TP	TN	FP	FN	AUC	Youden
#5	133.60 ± 8.10	137.70 ± 9.10	< 141.50	86.50%	37.60%	33.65%	89.36%	1.39	0.36	35	42	69	5	0.645	0.241
Platelet count ($x10^9$/L)															
#1			< 150	63.20%	65.60%	16.22%	94.40%	1.84	0.56	24	236	124	14		0.288
#15	135.00 ± 94.00	251.00 ± 87.00	< 150	62.00%	71.00%	52.00%	77.78%	2.14	0.54	13	28	12	8		0.330
#18	187.15 ± 118.47	285.19 ± 109.70	< 150	41.50%	96.15%	90.00%	67.53%	10.78	0.61	18	52	2	25	0.797	0.377
#23	220.00 ± 80.00	242.00 ± 83.00	< 150	41.00%	87.00%	85.71%	43.33%	3.15	0.68	24	26	4	34		0.280
#7	213.50<br>(10.00 - 417.00)	289.00<br>(162.00 - 416.00)	< 165	92.00%	68.00%	74.19%	89.47%	2.88	0.12	23	17	8	2	0.893	0.600
#9	109.00<br>(45.00 - 232.00)	400.00<br>(328.00 - 430.00)	< 278[c]	85.94%	90.91%	93.22%	81.63%	9.45	0.15	55	40	4	9	0.920	0.769
Mean platelet volume (fL)															
#10	9.56 ± 1.21<br>(9.06 - 10.06)	8.58 ± 0.83<br>(8.39 - 8.70)	> 9.50	56.00%	85.00%	76.60%	68.18%	3.73	0.52	36	60	11	28	0.734	0.410
#12	9.32	8.88	> 8.50	81.70%	51.20%	62.20%	72.73%	1.67	0.36	51	32	31	12	0.600	0.329
#13	10.30 ± 0.23	9.30 ± 0.19	> 9.95	65.30%	75.00%	72.22%	68.18%	2.61	0.46	13	15	5	7	0.730	0.403

Study No	Case Values[a]	Control Values[a]	Threshold	Sensitivity	Specificity	PPV	NPV	LR^+	LR^-	TP	TN	FP	FN	AUC	Youden
#18	9.97 ± 1.56	9.22 ± 1.44	> 9.00	63.40%	53.80%	51.92%	64.44%	1.37	0.68	27	29	25	16	0.641	0.172
#20	12.02 ± 1.54	9.42 ± 1.08	> 11.60[c]	77.80%	97.80%	97.22%	81.48%	35.36	0.23	35	44	1	10	0.890	0.756
#9	9.70 ± 1.30	8.90 ± 0.90	> 9.20	62.50%	72.73%	76.92%	57.14%	2.29	0.52	40	32	12	24	0.679	0.352
Platelet to lymphocyte ratio															
#14	15.00 ± 12.40 (6.20 - 22.00)	5.90 ± 3.50 (3.09 - 7.50)	> 7.00	70.00%	73.00%	71.79%	70.73%	2.59	0.41	56	58	22	24	0.780	0.430
#24	99.57 (69.61 - 128.57)	71.53 (53.98 - 88.29)	> 90.85	64.90%	80.00%	82.76%	60.61%	3.25	0.44	48	40	10	26	0.699	0.449
#3	62.40 ± 14.90	15.30 ± 2.10	> 57.70	91.30%	97.60%	96.83%	93.75%	38.04	0.09	61	90	2	6	0.847	0.889
